# Does liver cirrhosis affect the surgical outcome of primary colorectal cancer surgery? A meta-analysis

**DOI:** 10.1186/s12957-021-02267-6

**Published:** 2021-06-09

**Authors:** Yu-Xi Cheng, Wei Tao, Hua Zhang, Dong Peng, Zheng-Qiang Wei

**Affiliations:** Department of Gastrointestinal Surgery, The First Affiliated Hospital of Chongqing Medical University, Chongqing, 400016 China

**Keywords:** Liver cirrhosis, Colorectal cancer, Surgical outcome, Meta-analysis

## Abstract

**Purpose:**

The purpose of this meta-analysis was to evaluate the effect of liver cirrhosis (LC) on the short-term and long-term surgical outcomes of colorectal cancer (CRC).

**Methods:**

The PubMed, Embase, and Cochrane Library databases were searched from inception to March 23, 2021. The Newcastle-Ottawa Scale (NOS) was used to assess the quality of enrolled studies, and RevMan 5.3 was used for data analysis in this meta-analysis. The registration ID of this current meta-analysis on PROSPERO is CRD42021238042.

**Results:**

In total, five studies with 2485 patients were included in this meta-analysis. For the baseline information, no significant differences in age, sex, tumor location, or tumor T staging were noted. Regarding short-term outcomes, the cirrhotic group had more major complications (OR=5.15, 95% CI=1.62 to 16.37, p=0.005), a higher re-operation rate (OR=2.04, 95% CI=1.07 to 3.88, p=0.03), and a higher short-term mortality rate (OR=2.85, 95% CI=1.93 to 4.20, p<0.00001) than the non-cirrhotic group. However, no significant differences in minor complications (OR=1.54, 95% CI=0.78 to 3.02, p=0.21) or the rate of intensive care unit (ICU) admission (OR=0.76, 95% CI=0.10 to 5.99, p=0.80) were noted between the two groups. Moreover, the non-cirrhotic group exhibited a longer survival time than the cirrhotic group (HR=2.96, 95% CI=2.28 to 3.85, p<0.00001).

**Conclusion:**

Preexisting LC was associated with an increased postoperative major complication rate, a higher rate of re-operation, a higher short-term mortality rate, and poor overall survival following CRC surgery.

## Introduction

Liver cirrhosis (LC) is a common disease, causing 1.03 million deaths per year worldwide [1]. Alcohol misuse, infection with hepatitis viruses, and nonalcoholic liver disease are the leading causes of LC [2]. The pathological process of LC results from different mechanisms of liver injury, which leads to necroinflammation and fibrogenesis of liver tissues [3]. Moreover, the morbidity rate and the mortality rate are reported to be increased in patients with LC who undergo non-hepatic abdominal surgeries [4–6].

Colorectal cancer (CRC) is the third most common cancer and the second leading cause of cancer-related deaths worldwide [7]. Radical resection of the colorectal tumors has been widely accepted as a curative treatment [8]. Tumor location, comorbidity, clinical stage, anastomosis methods, and total mesorectal excision are reported to have an effect on the outcome of CRC surgery [9–12].

A recent study reported that LC had strong associations with the colorectal adenoma-carcinoma sequence [13]; however, the prognoses and outcomes of CRC surgery in patients with LC remain controversial.

Some studies reported that CRC patients with LC had more postoperative complications [14], but others reported the opposite [15]. In addition, few studies have assessed the specific surgical outcomes and perioperative management in patients with CRC. Thus, the purpose of this current meta-analysis was to evaluate the effect of LC on the short-term and long-term surgical outcomes of CRC.

## Methods

This study design stringently conformed to the Preferred Reporting Items for Systematic Reviews and Meta Analyses (PRISMA) statement [16]. The registration ID of this current meta-analysis on PROSPERO is CRD42021238042, and the link is https://www.crd.york.ac.uk/prospero/display_record.php?ID=CRD42021238042.

### Literature search

We conducted a systematic literature search of PubMed, Embase, and the Cochrane Library, and the last search was performed on March 23, 2021. The following keywords related to cirrhosis were used for the search: (liver cirrhosis) OR (cirrhosis) OR (cirrhotic). The items related to CRC were as follows: (colorectal cancer) OR (colon cancer) OR (rectal cancer) OR (colorectal neoplasm) OR (colon neoplasm) OR (rectal neoplasm) OR (colorectal tumor) OR (colon tumor) OR (rectal tumor). Then, we combined these items with “AND”. The search was restricted to publications in English, and two authors performed the search independently.

### Inclusion and exclusion criteria

Eligible studies were identified according to the following inclusion criteria: (1) human subjects and (2) studies reporting a comparison of surgical endpoints between cirrhotic patients and non-cirrhotic patients with CRC. The baseline information in the studies included the following: age, sex, tumor location, and tumor depth. Studies included the following primary or secondary endpoints as follows: major complications, minor complications, postoperative intensive care unit (ICU) admission, and the rate of re-operation or postoperative death. Postoperative complications were classified into two groups (minor complications and major complications) according to the Clavien-Dindo classification [17]. The exclusion criteria were as follows: (1) studies with incomplete data and (2) studies with no relevant endpoints. Case reports, case series, comments, letters to the editor, conference abstracts, and non-original articles were excluded.

### Study selection

All of the selected studies were screened by the same two authors. First, the titles and abstracts were screened to assess eligibility for inclusion in this meta-analysis. Then, the full texts were carefully checked to make final decisions based on the inclusion and exclusion criteria. For potential studies, an extensive manual search of relevant studies from the reference lists was conducted. Disagreements were resolved by a third senior author.

### Data extraction

Two authors extracted the data separately. The following data were collected: first author, year of publication, period of study, region of study, sample size, baseline information, major complications, minor complications, postoperative ICU admission, rate of re-operation, and postoperative death. The unpublished or unclear data were accessed by contacting the original authors through email if applicable. Then, the overall survival data were extracted by using the software Engauge Digitizer [18], with as many coordinate points taken as possible. Discussions were conducted in groups if disagreements occurred.

### Surgical outcomes

The surgical outcomes included the main outcomes and the secondary outcomes. The main outcomes of the current meta-analysis were postoperative complications, including minor complications and major complications. The secondary outcomes were postoperative ICU admission, the rate of re-operation, the short-term mortality rate, and long-term survival.

### Quality assessment

The Newcastle-Ottawa Scale (NOS), which has a score ranging from zero to nine points, was used to assess the quality of the enrolled studies. Three domains, selection, comparability, and results, were evaluated for each study [19]. A study with a score of nine points was considered high quality, a study with a score of seven to eight points was considered medium quality, and a study with six or less was considered low quality. The senior author completed this assessment independently.

### Statistical analysis

The mean differences (MDs) and 95% confidence intervals (CIs) were calculated for age. The odds ratios (ORs) and 95% CIs were calculated for sex, tumor location, tumor depth, and surgical outcomes. The statistical heterogeneity for the included studies was evaluated by using the I^2^ value. The random effects model was used when I^2^>50%, which was considered to indicate high heterogeneity, and p<0.1, which was considered to indicate statistical significance. Otherwise, the fixed effects model was used, and p<0.05 was considered statistically significant [20]. RevMan 5.3 (The Cochrane Collaboration, London, UK) was used to perform the data analysis in this meta-analysis.

## Results

### Study selection

**Fig. 1:**
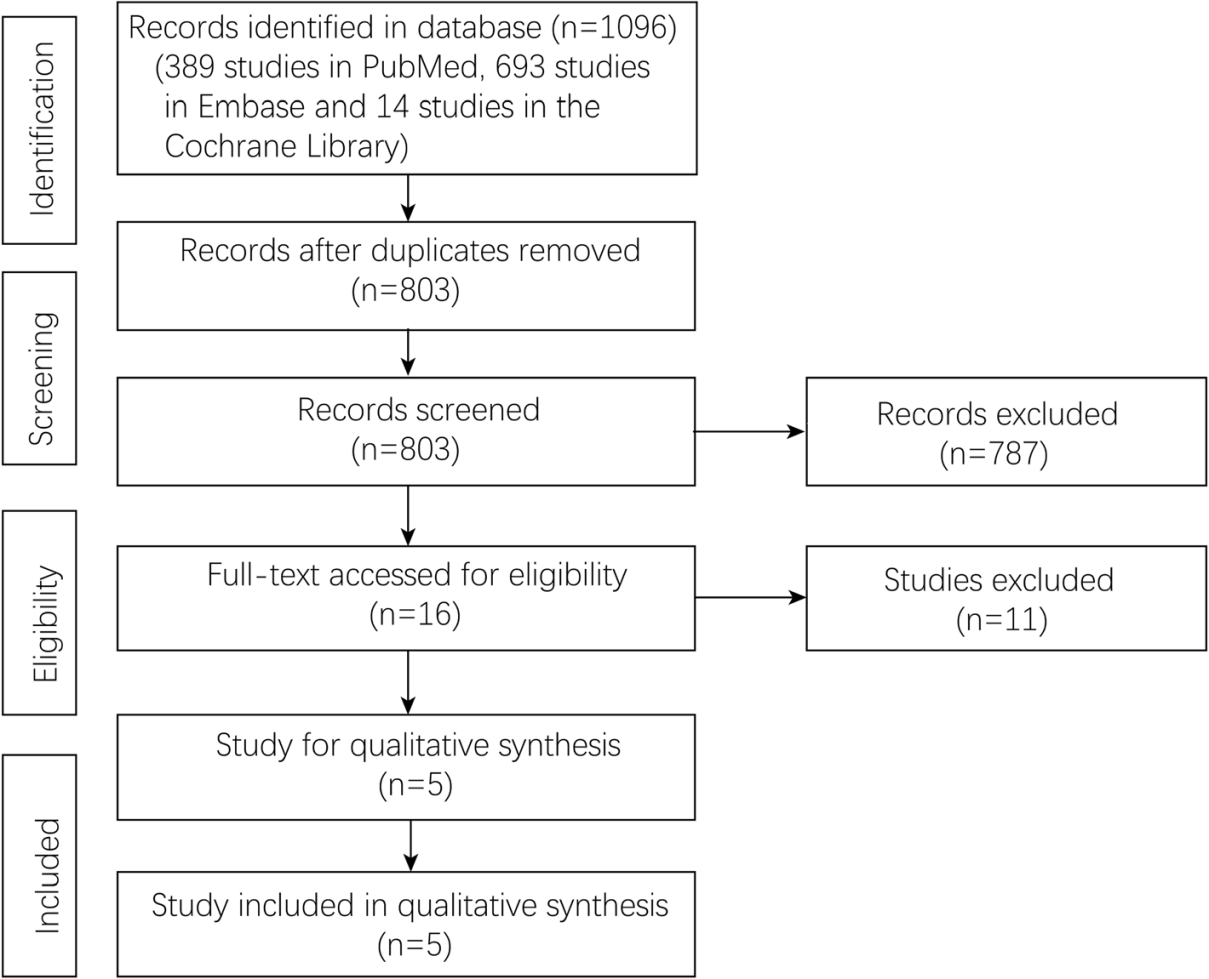
Flowchart of study selection

From an initial total of 1096 studies identified in the databases (389 studies in PubMed, 693 studies in Embase, and 14 studies in the Cochrane Library), 293 were removed due to duplication. After screening the titles and abstracts, 16 studies underwent a full-text review. Finally, five studies [4, 14, 15, 21, 22] were included in this meta-analysis. The flow chart of article selection is shown in Fig 1.

### Patient characteristics and quality assessment of the included studies

In total, five studies with 2485 patients were included in this meta-analysis. All studies were retrospective studies from diverse regions, including Romania, France, Denmark, and Korea. The year of publication ranged from 2013 to 2020, and the study date ranged from 1996 to 2014. The grade of complications and the NOS score are shown in Table 1.

**Table 1:** Characteristics of the studies included in the meta-analysis

Author	Year published	Country	Study design	Study date	Sample size		Postoperative complications Clavien-Dindo classification (I/II/III/IV/V)		NOS
					Cirrhotic group	Non-cirrhotic group	Cirrhotic group	Non-cirrhotic group	
Shin et al. [15]	2020	Korea	Retrospective	2008–2013	453	906	II/III	II/III	8
Lacatus et al. [21]	2018	Romania	Retrospective	2005–2014	68	136	I/II/III/IV/V	I/II/III/IV/V	7
Han et al. [4]	2017	Korea	Retrospective	2002–2010	55	220	Unknown	Unknown	7
Sabbagh et al. [14]	2016	France	Retrospective	2006–2014	40	80	Unknown	Unknown	7
Montomoli et al. [22]	2013	Denmark	Retrospective	1996–2009	158	369	III/IV/V	III/IV/V	8

Abbreviations: *NOS* Newcastle-Ottawa Scale

### Baseline information

Age, sex, tumor location, and tumor depth were extracted as baseline information. The results showed no significant differences in sex (OR=1.28, 95% CI=0.92 to 1.79, I^2^=54%, *p*=0.14), age (MD=0.06, 95% CI=−0.93 to 1.05, I^2^=0%, *p*=0.91), tumor location (colon: OR=0.82, 95% CI=0.62 to 1.09, I^2^=0%, *p*=0.17; rectal: OR=1.21, 95% CI=0.91 to 1.61, I^2^=0%, *p*=0.18), or tumor T staging (T1-T2: OR=1.14, 95% CI=0.77 to 1.69, I^2^=69%, *p*=0.52; T3-T4: OR=0.81, 95% CI=0.59 to 1.12, I^2^=53%, *p*=0.20) between the cirrhotic group and non-cirrhotic group (Table 2).

**Table 2:** Summary meta-analysis of comparison between cirrhotic group and non-cirrhotic group

Subgroup	Studies	Participants (cirrhotic/non-cirrhotic)	Odds ratio/mean difference (95% CIs)	Heterogeneity
Baseline information				
Age, year	2	521/1042	0.06 (−0.93, 1.05); p=0.91	I^2^=0%; p=0.78
Male	5	774/1711	1.28 (0.92, 1.79); p=0.14	I^2^=54%; p=0.07
Tumor location colon	4	321/805	0.82 (0.62, 1.09); p=0.17	I^2^=0%; p=0.61
Tumor location rectal	4	321/805	1.21 (0.91, 1.61); p=0.18	I^2^=0%; p=0.64
Tumor staging				
T1-T2	4	706/1575	1.14 (0.77, 1.69); p=0.52	I^2^=69%; p=0.02
T3-T4	4	706/1575	0.81 (0.59, 1.12); p=0.20	I^2^=53%; p=0.09

Abbreviations: *95% CIs* 95% confidence intervals

### Complications

**Fig. 2:**
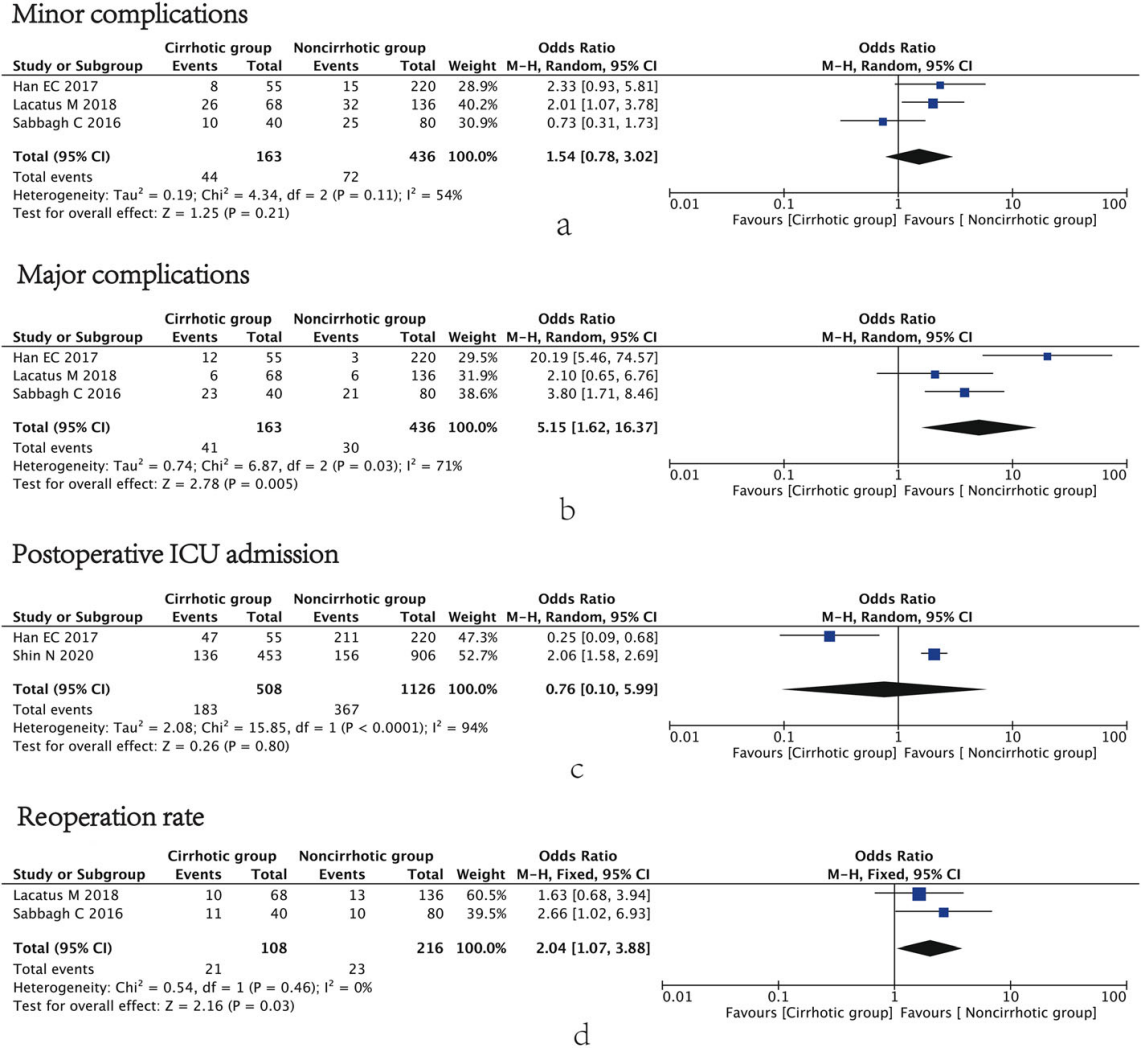
Forest plot showing the short-term outcomes. **a** Minor complications, **b** major complications, **c** postoperative ICU admission, and **d** Reoperation rate

Three studies [4, 14, 21] investigated minor complications, and no significance was observed between the two groups (OR=1.54, 95% CI=0.78 to 3.02, I^2^=54%, *p*=0.21) (Fig. 2a). However, in terms of major complications in the same three studies [4, 14, 21], the cirrhotic group had more major complications than the non-cirrhotic group (OR=5.15, 95% CI=1.62 to 16.37, I^2^=71%, *p*=0.005) (Fig. 2b).

### Postoperative ICU admission

Two studies [4, 15] including 1634 participants reported postoperative ICU admission. The results indicated no difference between the cirrhotic group and non-cirrhotic group (OR=0.76, 95% CI=0.10 to 5.99, I^2^=94%, *p*=0.80) (Fig. 2c).

### Reoperation rate

Two studies [14, 21] investigated the rate of re-operation, and the cirrhotic group showed a higher rate of re-operation than the non-cirrhotic group (OR=2.04, 95% CI=1.07 to 3.88, I^2^=0%, p=0.03) (Fig. 2d).

### Short-term mortality rate

**Fig. 3:**
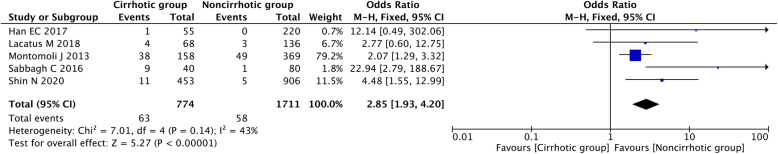
Forest plot showing the short-term mortality rate

All five studies [4, 14, 15, 21, 22] including 2485 patients reported the short-term mortality rate. The cirrhotic group had a higher death rate than the non-cirrhotic group (OR=2.85, 95% CI=1.93 to 4.20, I^2^=43%, *p*<0.00001) (Fig. 3).

### Long-term survival

**Fig. 4:**
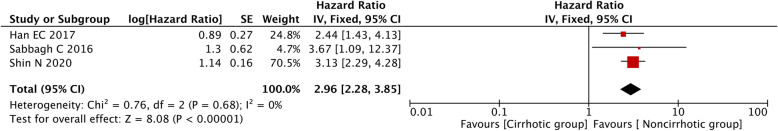
Forest plot showing the long-term survival

Three articles [4, 14, 15] reported long-term survival. The non-cirrhotic group had a better overall survival than the cirrhotic group (HR=2.96, 95% CI=2.28 to 3.85, I^2^=0%, *p*<0.00001) (Fig. 4).

### Sensitivity and publication bias

**Fig. 5:**
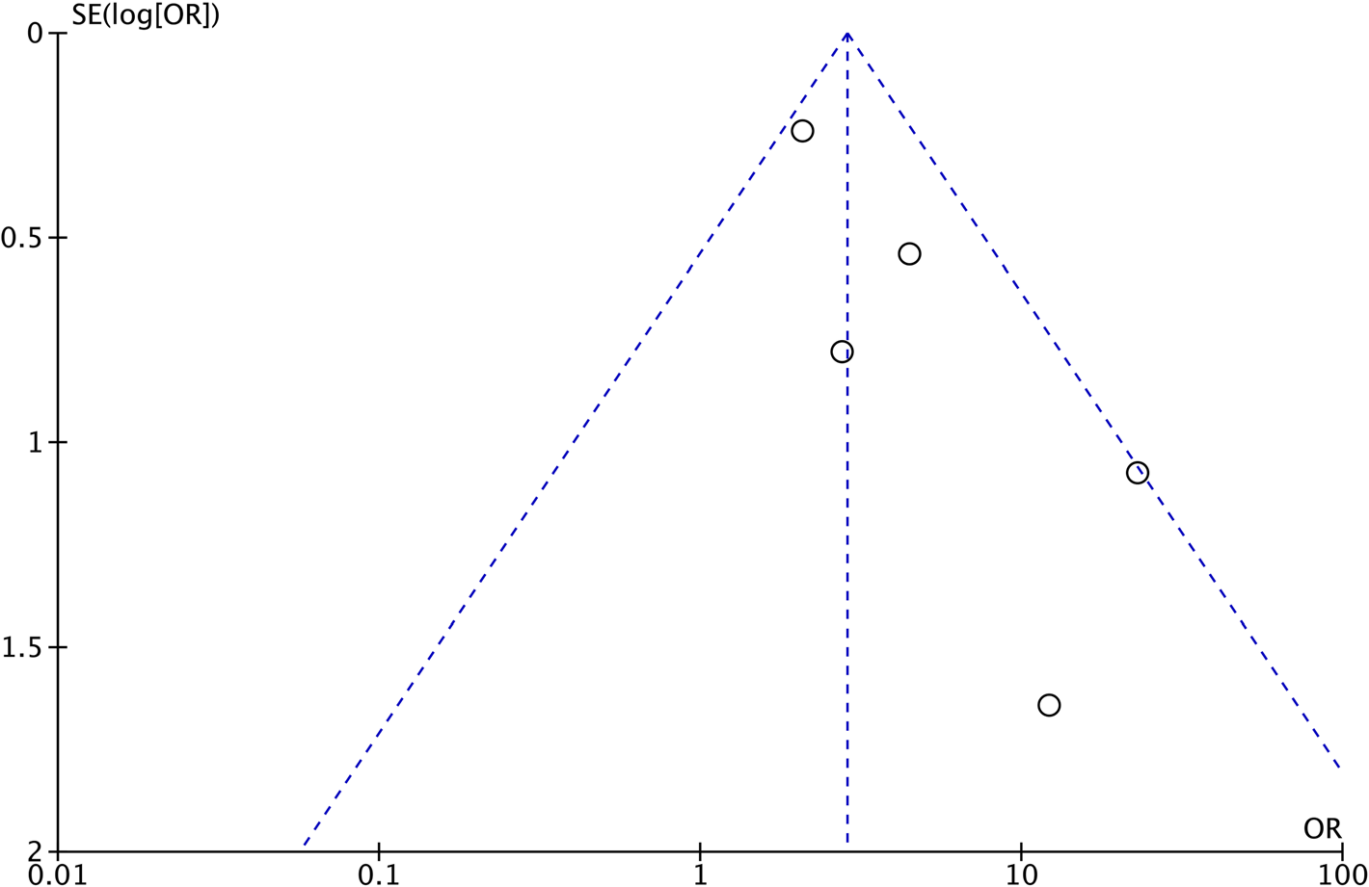
Funnel plot showing the short-term mortality rate

Repeated meta-analysis was performed by excluding one study at a time, and the exclusion of any one study did not significantly alter the results. Publication bias for the included studies was based on a visual inspection of the funnel plot. The funnel plot was symmetrical, and no obvious publication bias was found (Fig. 5).

## Discussion

LC is a serious comorbidity in cancer-related diseases. Previous studies found that LC had a negative effect on CRC surgery [14, 15]. In this meta-analysis, five studies with 2485 patients were included. Regarding baseline information, no significant difference was found in terms of age, sex, tumor location, or tumor T staging between the two groups. Regarding short-term outcomes, the cirrhotic group had more major complications, a higher rate of re-operation, and a higher short-term mortality rate than the non-cirrhotic group. However, minor complications and the ICU admission rate did not significantly differ between the two groups. Moreover, the non-cirrhotic group showed a longer survival time than the cirrhotic group.

LC is a chronic disease with many complications, including variceal bleeding, ascites, hepatorenal syndrome, hepatic encephalopathy, and hepatocellular carcinoma in the decompensated stage [23]. Patients with LC suffer from economic burdens and a shortened life expectancy [24, 25]. A high risk of LC was noted among patients with cirrhosis undergoing abdominal surgery [26]; however, LC was not a contraindication for abdominal surgery or chemotherapy [27]. The preoperative albumin level is correlated with surgical outcomes related to refractory ascites [28]. Previous studies reported that patients with LC undergoing gastrectomy had more complications and a higher mortality rate short term [29, 30]. In this meta-analysis, the cirrhotic group had more major complications than the non-cirrhotic group after CRC surgery. However, no significant difference in minor complications was noted between the two groups. The reasons for this might be the reduced recuperative capacity of the patients, reduced drug metabolism in the liver, and weakened immune function [29–31]. In addition, more major complications could cause a higher rate of re-operation, which is consistent with the results of this meta-analysis. The reason for the higher rate of re-operation in CRC patients with LC might be the inadequate preoperative adjustment of liver function. Hyperbilirubinemia, prothrombin time prolongation, and intraoperative transfusion have been reported as risk factors for postoperative complications in these patients [32]. Therefore, the perioperative management of LC patients who undergo surgery for CRC is crucial.

CRC has already become the second most common cancer-related disease worldwide [7], and patients with concurrent LC and CRC might have a higher death rate after surgery. Previous studies showed that the mortality of the LC group ranged from 18 to 26%, which was higher than that of the non-LC group, and the differences among studies might be due to the patients being treated at community hospitals or specialized hospitals [33, 34]. We observed that the cirrhotic group had a higher short-term mortality rate and a shorter survival time than the non-cirrhotic group in this meta-analysis. A possible reason for finding is that decreased liver function could cause hepatic coagulopathy, lower albumin levels, and abnormal liver metabolism, which led to more severe complications, including ascites, infection, bleeding, and anastomotic fistulas [5]. The major complications might be related to short-term death after CRC surgery. Moreover, cirrhosis and intestinal dysfunction cause chronic malnutrition, and LC patients have a high risk for primary hepatic carcinoma [2], which might influence long-term survival.

Before patients with confirmed or suspected LC undergo CRC surgery, a preoperative assessment of liver function is needed to clarify its severity, which should be evaluated by either the Child-Pugh classification (CTP) or Model for End Stage Liver Disease (MELD) score in clinical practice [35, 36]. A previous study compared CRC patients with different CTPs in terms of surgical outcomes and found that Child B patients had a high rate of complications, more reinterventions, and a longer hospitalization stay [21]. Although classification or risk stratification is vital before surgery in CRC patients with LC, it was lacking in the included studies. The lack of a liver function assessment limited the ability to guide preoperative decisions regarding who underwent CRC surgery, which would require knowing the severity of cirrhosis. Thus, studies on CRC patients with LC should be more comprehensive and include the CTP or MELD score.

There were several strengths of our meta-analysis. First, this meta-analysis was the first to exclusively pool all of the data to evaluate the effect of LC on the short-term and long-term surgical outcomes of CRC. Second, the controversy about surgical outcomes and long-term survival between the cirrhotic patients and non-cirrhotic patients was settled in this meta-analysis. Third, the present meta-analysis provided some information that will be useful in clinical practice. Perioperative management should be handled cautiously by surgeons among patients with LC, and furthermore, patients should be cautious during the postoperative period due to poor overall survival.

However, certain limitations to this current meta-analysis existed. First, only five studies were included, all of which were retrospective studies; however, no publication bias was shown in the funnel plot. Second, cirrhosis was not classified in each study, and compensated LC and decompensated LC might have different outcomes after CRC surgery. Third, liver function was graded by the CTP or MELD score in only one study, which might have influence on the accuracy of the results. Finally, the long-term survival was analyzed with data from only three studies, which might have increased the clinical heterogeneity. Therefore, comprehensive, prospective, and high-quality randomized controlled trials should be performed in the future.

In conclusion, preexisting LC was associated with an increased postoperative major complication rate, a higher rate of re-operation, a higher short-term mortality rate, and poorer overall survival following CRC surgery. Therefore, surgeons should be careful when conducting CRC surgery on patients with LC.

## Data Availability

All data generated or analyzed during this study are included in this published article.

## Abbreviations

LC: Liver cirrhosis
CRC: Colorectal cancer
ICU: Intensive care unit
NOS: Newcastle-Ottawa Scale
PRISMA: Preferred Reporting Items for Systematic Reviews and Meta Analyses
CTP: Child-Pugh classification
MELD: Model for End Stage Liver Disease
